# Effect of 3D-Printed PLA Structure on Sound Reflection Properties

**DOI:** 10.3390/polym14030413

**Published:** 2022-01-20

**Authors:** Katarina Monkova, Martin Vasina, Peter Pavol Monka, Jan Vanca, Dražan Kozak

**Affiliations:** 1 Faculty of Manufacturing Technologies, Technical University in Kosice, 080 01 Presov, Slovakia; peter.pavol.monka@tuke.sk (P.P.M.); jan.vanca@tuke.sk (J.V.); 2 Faculty of Technology, Tomas Bata University in Zlin, Nam. T.G. Masaryka 275, 760 01 Zlin, Czech Republic; 3 Faculty of Mechanical Engineering, VŠB-Technical University of Ostrava, 17. listopadu 15/2172, 708 33 Ostrava-Poruba, Czech Republic; 4 Mechanical Engineering Faculty, University of Slavonski Brod, Trg Ivane Brlic-Mazuranic 2, HR-35000 Slavonski Brod, Croatia; dkozak@unisb.hr

**Keywords:** polylactic acid, sound reflection, excitation frequency, porosity, 3D printing technique, thickness, air gap

## Abstract

3D printing technique is currently one of the promising emerging technologies. It is used in many areas of human activity, including acoustic applications. This paper focuses on studying the sound reflection behavior of four different types of 3D-printed open-porous polylactic acid (PLA) material structures, namely cartesian, octagonal, rhomboid and starlit structures. Sound reflection properties were evaluated by means of the normal incidence sound reflection coefficient based on the transfer function method using an acoustic impedance tube. In this study, various factors affecting the sound reflection performance of the investigated PLA samples were evaluated. It can be concluded that the sound reflection behavior of the tested PLA specimens was strongly affected by different factors. It was influenced, not only by the type of 3D-printed open-porous material structure, but also by the excitation frequency, the total volume porosity, the specimen thickness, and the air gap size behind the tested specimen inside the acoustic impedance tube.

## 1. Introduction

Architects have often focused on designing beautiful buildings intended for musical performances, especially concert halls, operas, churches, mosques, or theatres. Acoustic design with good sound distribution is crucial in the internal spaces of such buildings [1,2,3]. In contrast to concert halls, in which the best possible sound acoustics are needed, in the case of main roads and other places, where there is high noise pollution, the aim is to absorb or effectively reflect noise, thus preventing the further propagation of sound waves [4]. However, other rooms which are used every day, such as classrooms, auditoriums, offices, or production halls, also require well-projected sound properties. Poor acoustic design of such workspaces can cause discomfort, dizziness, and constant exposure to excessive unwanted sounds that affect physical and mental health. Therefore, it is crucial for human health and safety to use suitable sound-absorbing materials in specific rooms, offices, manufacturing halls, vehicles, or aircraft.

Noise and sound have the same specification but are perceived differently. The speed of sound can vary due to the difference in pressure between the media through which the sound propagates [5]. Sound can be considered as the propagation of a disorder mainly in liquid or solid phases. The wave motion is triggered when the element moves the nearest air particle, which then causes a pressure difference in the medium in which the wave moves [6]. Noise is perceived as a nuisance by people, and noise pollution in the environment can also cause discomfort [7].

Sound propagates through the air or other media as a longitudinal wave, in which the mechanical vibrations run in the direction of wave propagation. The property of waves and sound associated with the echo phenomenon is known as reflection [7,8,9,10].

The sound wave changes during the passage through space when it meets an obstacle. The wave may vary due to reflection from the obstacle, diffraction around the obstacle, and transmission accompanied by a refraction to the obstacle and other space. One part of the soundwave passes through the boundary of space. The second part is reflected. The reflection itself and its intensity depend on the similarity of the spaces [11].

The sound energy reflected from a boundary/obstacle can be divided into specular or diffuse. The former is when the reflection appears at the same angle at which it strikes the surface, like the reflection of light from a mirror. In contrast, diffuse reflection occurs when the sound energy is scattered in non-specular directions [4]. The direction of wave propagation is perpendicular to the front formed by all the Huygens’ wavelets. In terms of shape, suitable reflectors are used for different purposes or effects. For example, a parabolic reflector focuses a parallel sound wave to a specific point, making it easier to hear a feeble sound. Such reflectors are used in parabolic microphones to pick up sound from a distant source or select the location where the sound is observed and then focus it on the microphone. On the other hand, an elliptical shape can focus sound from one point to another [8,12].

The reflection (absorption) of sound waves is affected by several parameters, such as surface shapes, obstacles and their spatial distribution, their distance and arrangement [13]. In addition to these, one of the most important factors that has a significant effect on the proper propagation of sound is the material, including its porosity, density, thickness, and the angle of sound incidence, surface shape, excitation frequency of the acoustic wave and the type of internal structure of the material [1,6]. Zulkifli [14] and Lee [15] reported that the acoustic absorption of multilayer materials is better with perforated plates backed with airspaces. In most cases, sound-absorbing materials are measured in a reverberation chamber. In a study by Cucharero et al. [16], the authors showed how angle-dependent absorption coefficients could be measured. The results were in good agreement with sound absorption coefficients and confirmed that materials have different attenuation behavior to sound waves coming from different directions.

In recent years, with the development of manufacturing technology, porous materials have presented a challenge to many researchers to study their sound absorption or reflection properties. The process of reflecting acoustic waves in porous materials in the case of oblique impact of acoustic waves was studied by Gimaltdinov et al. [17] The results showed, that with increasing angle of impact, transmission decreases. According to [18], lightweight materials, such as foam or fabric, are often too porous to reflect sound passing through, and their energy is converted into heat with a reduction in magnitude. This approach is often used in cinemas and recording studios to reduce the room’s reverberation time. While effective internally, lightweight materials are not suitable externally, so concrete is still preferred [18]. A balance between speed and density of sound and its effect on the acoustic properties of a porous material was studied by Kuczmarski et al. [19], while the effect of tortuosity, porosity, and flow resistivity on reflection and transmission of acoustic waves in porous materials was the topic of [20]. Sun et al. [21] demonstrated that the main reason for the difference in acoustic properties of materials produced via selective laser sintering is the difference in the unit structures. The investigation confirmed that transmission loss has better results with mixed structures than using individual components, and can be improved with periodically arranged scatterers inside porous materials.

To study the natural behavior of the uniform infinite layers of a porous material was the aim of [22], which considered the relation between pressure and velocity. The results of this study enabled evaluation of the intrinsic properties of the material based on plane wave acoustic surface impedance. A subsequent comprehensive literature review, with detailed description of the prediction of acoustic absorption behavior, various techniques for enhancing sound absorption and the application of Wilson’s relaxation-matched equivalent fluid model, was provided by Otaru [23]. The compound impedance and sound absorption in a porous material in the first resonance cavity were examined by Fan [24]. The results showed that an acoustic board, consisting of aluminum foam as the inner structure and micro-porous board as the outer structure, with polyester between them, showed good sound absorption performance in the low-frequency zone. Similarly, Talebitooti et al. [25] investigated double-walled poroelastic composite shells to achieve diffuse sound transmission in the high-frequency range. A positive effect was observed in optimizing vibroacoustic fitness.

A summary of fabrication, evolution and prediction models for sound absorption in porous materials is described in the work of Cao et al. [26], while the work of Chen [27] demonstrated the relation between geometrical parameters and sound absorption based on the establishment of static flow resistivity and derivation of the fractal acoustic model. The finite analysis method was applied to assess sound insulation performance in a sandwich plate with lattice core, the results showing that a sandwich plate with a lattice core could outperform traditional lattice cores [28].

The present study represents an original contribution to the research of sound properties of additively produced materials with regularly distributed porous structures. It sought to investigate the sound reflection behavior of four different types of 3D-printed open-porous polylactic acid (PLA) material structures, namely cartesian, octagonal, rhomboid and starlit structures, considering various factors affecting the sound reflection performance of the investigated PLA samples. The sound reflection properties were evaluated by means of the normal incidence sound reflection coefficient based on the transfer function method using an acoustic impedance tube.

It can be seen from the overview above that the concept of using lightweight materials in technical practice is not entirely new. Porous materials give the product a specific combination of properties, especially in terms of lightness (weight reduction), while maintaining sufficient rigidity, strength, and resistance to load/stress. Their advantages include, not only their mechanical properties, but also other physical characteristics, such as acoustic, thermal, or viscous properties. Reduced material consumption will also reduce the cost of producing the component and recycling it, making porous materials more environmentally friendly. Currently, the production of cellular materials is possible mainly due to the rapid development of additive technologies. With the help of 3D printing technique, it is possible to produce even complex objects that cannot be produced by other (classical) methods. All porous materials are strongly dependent on their three-dimensional (3D) microstructures, including the porosity, pore sizes, shapes, and connectivity of the porous space [29,30]. In terms of the division into porous structures, regular and irregular, the regular arrangement of cellular structures allows better control or prediction of their properties, so the authors decided to address this type of structure in the sound reflectivity research.

To the best of the author’s knowledge, no relevant studies have yet been published which examine the sound reflection of the above types of structures made of PLA plastic material, considering the influence of several factors, such as their structural type, excitation frequency, the total volume porosity, the specimen thickness, and the air gap size behind the tested specimen inside the acoustic impedance tube.

## 2. Materials and Methods

### 2.1. Characteristics of Samples

Airflow resistivity is a physical parameter characteristic of porous and fibrous materials, which quantifies, per unit length, the ability to oppose resistance to the motion of air particles inside a material. As is well-known, this property is important to evaluate the acoustic behavior of these materials in various fields of their application, such as automotive noise mitigation, architectural acoustics, and building acoustics [31,32].

The samples were produced via an additive approach from polylactic acid or polylactide (PLA) material (Prusa Polymers, Prague, Czech Republic), which is a thermoplastic made from vegetable starch. The processing of this material was carried out at a temperature of approximately (180–220) °C. The manufacturer states in the material sheet the following properties of the PLA filament used in the experiments of this research: peak melt temperature (145–160) °C, glass transition temperature (55–60) °C, specific weight 1.24 g cm^−3^, moisture absorption 0.3% at 28 °C and yield strength of the filament 57.4 MPa.

After heating this material, it is possible to smell a sweet scent. PLA material is resistant to deformation, non-toxic, brittle, and less heat resistant than ABS material. The advantage of this material is that it is not harmful to health and is biodegradable. Another advantage of this material is that it cannot be easily torn and can be used with printers that do not have a heated printing bed. Compared to other materials, PLA cooling takes longer, and efficient cooling can be ensured by fans. Components made of this material can be finely machined, e.g., by grinding. Products extruded from this plastic should not be exposed to direct sunlight. The products are not suitable for high loads. In situations of excessive humidity, these products absorb water, which also reduces their quality. PLA material is most often used to produce food aids, prototypes, toys, design models, etc. However, it can be used to advantage in suitable technical applications in engineering and construction, and the automotive or aerospace industries [33,34].

Four different types of 3D-printed open-porous structures, namely, cartesian, octagonal, rhomboid and starlit structures, were selected to investigate sound reflection properties within the presented research. The main reason for selecting the lattice structures referred to above was the completely different type of regularly repeating cells forming the structure, as well as the nature of their distribution in the cross-sectional area (rotated around the cylindrical axis or slid in rectangular axes). Virtual models of the samples were generated in software PTC Creo 7 (Parametric Technology Corporation Inc., Boston, MA, USA). The samples were cylindrical with a diameter of 29 mm so that their outer shape and dimensions matched the impedance tube for testing; the structure itself had a diameter of 25 mm, and the outer shell of continuous material had a thickness of 2 mm. For each type of structure (cartesian, octagonal, rhomboid and starlit), samples were produced in three different volume porosities *P* (30%, 43% and 56%) and in three different thicknesses *t* (10 mm, 20 mm and 30 mm), making in total 36 pieces.

The total volume porosity of a porous material is one of the essential characteristics of a structure (apart from its geometry) and is defined by Equation (1) [35]:

(1) $$P\left(\%\right)=\frac{V_{a}}{V_{T}}\times 100$$

where *V_a_* is the air volume in body cavities, and *V_T_* is the total body volume. The volume porosity of the specimens was regulated by the thickness of the strut in a lattice structure and by the cell size.

If the apparent density of the samples is known (in our case it was 0.5456; 0.7068 and 0.868 gcm^−3^), the porosity can be expressed by means of Equation (2)

(2) $$P\left(\%\right)=\left(1-\frac{\rho_{A}}{\rho }\right)\times 100$$

where *ρ_A_* is the apparent density and *ρ* is the density of PLA material (1.24 gcm^−3^).

A summary of the basic characteristics of lattice structures made to investigate the sound reflection properties is given in Table 1, where *x/y/z* are the dimensions of the base cell, where the orientation “*z*” is the direction of sample building and it is perpendicular to the position of the platform “*x,y*”.

The ability to produce such complex structures in the core of an object (e.g., a future sound wall with the necessary sound reflection properties) is made possible today by technologies which work on the additive principle. The proposed test specimens were printed on a Prusa i3 Mk2 3D printer (Prusa Research a.s., Prague, Czech Republic), using fused deposition modeling (FDM) technology for 3D printing. This technology is one of the most widespread additive production technologies and is included in the group of methods used to produce solid prototypes based on the principle of extrusion. As with other technologies, including FDM, the required body is created by depositing thin layers of semi-liquid material. The material is wound in the form of a filament on a spool from where it is led to a nozzle located in the application head. The movement of the nozzle is given by a 3D model of the desired body, which is sliced into thin layers in the software before the 3D printing itself. It is “controlled” by the contours of the body in the given layer and the chosen strategy of filling continuous surfaces with the material. After application, the material adheres to the previous layer and gradually cools. The building platform, on which the body is built, drops lower with increasing layer thickness and the application process is repeated until the component is completed. The quality and strength of FDM components essentially depend on the process parameters [36,37]. The material is wound on a spool attached to the extruder with its motor, which pushes the thread through the hot end. The hot end maintains a constant temperature to melt the filament very quickly into a viscous liquid which is extruded through a small nozzle, firstly onto the platform, and then in subsequent layers, continuing to apply the material until the entire prototype is formed.

The 3D printing strategy, as well as the nozzle and platform movement, were controlled by a computer on which PrusaSlicer software of the same manufacturer as the 3D printer machine (Prusa Research a.s., Prague, Czech Republic) was installed.

**Figure 1:**
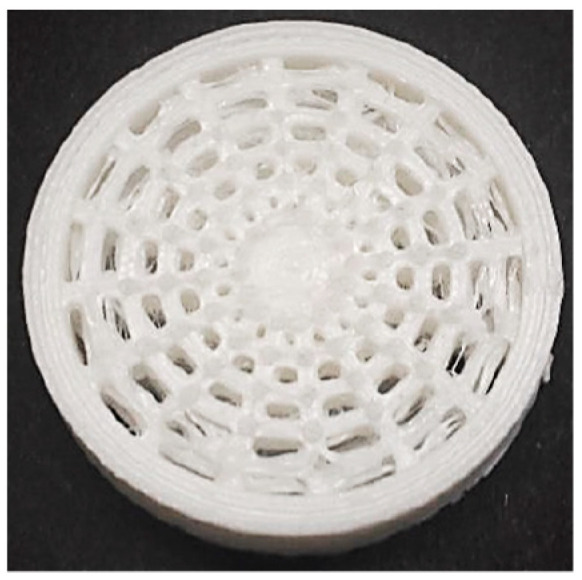
The 3D printer stringing effect appearance of a preliminary sample.

The technical and technological parameters used in 3D printing were selected according to the equipment manufacturer’s recommendations for PLA material. However, a range of temperatures and speeds could be used. A nozzle temperature of 220 °C and a print speed of 40 mms^−1^ for the whole sample were applied to produce a preliminary sample. In this case, the 3D printer stringing effect appeared as seen in Figure 1.

After several trials to adjust the settings, the following 3D printing parameters were finally selected: nozzle temperature 215 °C for the first layer and 210 °C for the others; bed temperature (building platform) 60 °C, printing speed on the peripheral circuits 30 mm/s and printing speed of the internal structure 20 mms^−1^. A 1.7 mm diameter filament was used for 3D printing of the samples, which was applied in layer thickness of 0.254 mm using a 0.4 mm diameter nozzle. The samples were positioned so that their cylindrical axes were perpendicular to the building platform. They were built without supports because the lattice structures were self-supporting. The quality of the samples was checked visually, and no cracks or thin strands of plastic (as the result of 3D printer stringing) were observed.

**Figure 2:**
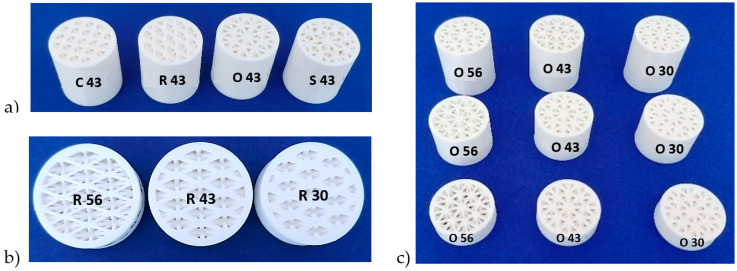
Examples of samples produced. (**a**) samples of thickness *t* = 30 mm and volume porosity *P* = 43% with different types of structure, (**b**) rhomboid samples with different volume porosities and (**c**) a set of samples with octagonal structure. (Note: the numbers on the sample labels in the figure indicate the total volume porosity in %).

An example of the samples produced is shown in Figure 2. In Figure 2a, samples of thickness *t* = 30 mm with different types of structure are shown. In Figure 2b rhomboid samples with different volume porosities are shown, and in Figure 2c a set of samples with octagonal structure is presented.

### 2.2. Measurement Methodology

#### 2.2.1. Sound Reflection Coefficient

When sound propagates from a sound source to a material’s surface, the incident sound energy *E_I_* is either reflected or absorbed by this material [38,39]. The sound reflection coefficient *β* is the measure of how much sound is reflected from a material [40]. It can be expressed as follows [41,42,43,44]:

(3) $$\beta =\frac{E_{R}}{E_{I}}=\frac{E_{I}-E_{A}}{E_{I}}=1-\alpha$$

where *E_R_* is the reflected sound energy, *E_A_* is the absorbed sound energy, and *α* is the sound absorption coefficient.

#### 2.2.2. Sound Reflection Properties

There are two different standardized methodologies for measuring sound reflection properties of material specimens, namely, the reverberation room and impedance tube methods [45].

Measurement of sound reflection properties using the reverberation room method [46,47,48] is performed in a standardized reverberation chamber and is based on the measurement of reverberation times with and without the specimen to be tested. This method is preferred by materials producers to measure the random sound reflection coefficient in a diffuse sound field. However, the reverberation room method requires a large-scale chamber of volume *V* > 200 m^3^ and a large surface area (i.e., 10–12 m^2^) of investigated specimens.

The impedance tube method [49,50] measures the sound reflection coefficient when sound waves propagate vertically through a test sample. Compared to the reverberation room method, the impedance tube method is a quick, low-cost method, using small-size specimens. Therefore, this method is suitable for developing new types of soundproofing materials. There are three impedance tube methods: pipe pulse, standing wave ratio, and transfer function methods [49]. The pipe pulse method is based on the separation of incident and reflection acoustic signals. Sound reflection properties are subsequently determined from the amplitude ratio of the acoustic signals. This method requires high anti-reference and capability of signal separation. The standing wave method [51] consists in measuring acoustic pressure amplitudes of the incident and reflected acoustic waves by means of a movable acoustic probe. This method is tedious, and the measuring efficiency is relatively low. The transfer function method [52] uses two precisely matched microphones and is based on a similar principle to the standing wave method. Its operational process is much simpler, and the measuring efficiency is higher than the standing wave method [49].

**Figure 3:**
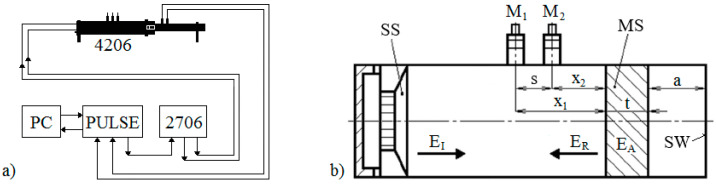
Circuit diagram of the experimental equipment for measuring the sound reflection coefficient (**a**) and a schematic of the acoustic impedance tube (**b**). Legend to the abbreviations: *a*—air gap size; *E_A_*—absorbed sound energy; *E_I_*—incident sound energy; *E_R_*—reflected sound energy; M_1_, M_2_—measuring microphones; MS—measured specimen; *s*—distance between microphones M_1_ and M_2_; SS—sound source; SW—solid wall; *t*—sample thickness; *x*_1_, *x*_2_—microphone distances from the tested PLA sample surface.

Experimental measurements of sound reflection properties of the investigated PLA specimens were undertaken using the transfer function method ISO 10534-2 [52] that is based on measuring sound pressures in the impedance tube by means of microphones M_1_ and M_2_. This method defines the normal incidence sound reflection coefficient *β* as follows [53,54,55]:

(4) $$\beta ={\left|R\right|}^{2}$$

where *R* is the complex reflection factor that is given by the formula:

(5) $$R=\frac{H_{12}-H_{I}}{H_{R}-H_{12}}e^{2jk_{0}x_{1}}$$

where *H*_12_ is the pressure transfer function, *H_I_* is the transfer function for the incident acoustic wave, *H_R_* is the transfer function for the reflected acoustic wave, *k*_0_ is the complex wave number, and *x*_1_ is the distance between the microphone M_1_ and the tested material sample (see Figure 3b). The above transfer functions are expressed as:

(6) $$H_{12}=\frac{p_{2}}{p_{1}}=\frac{e^{jk_{0}x_{2}}+R\cdot e^{-jk_{0}x_{2}}}{e^{jk_{0}x_{1}}+R\cdot e^{-jk_{0}x_{1}}}$$

(7) $$H_{I}=e^{-jk_{0}\left(x_{1}-x_{2}\right)}=e^{-jk_{0}s}$$

(8) $$H_{R}=e^{jk_{0}\left(x_{1}-x_{2}\right)}=e^{jk_{0}s}$$

where *p*_1_ and *p*_2_ are the complex acoustic pressures measured by the microphones M_1_ and M_2_, *x*_2_ is the distance between the microphone M_2_ and the studied material specimen, and *s* is the distance between the two microphones M_1_ and M_2_.

The complex acoustic pressures are given by the equations:

(9) $$p_{1}={\hat{p}}_{I}e^{jk_{0}x_{1}}+{\hat{p}}_{R}e^{-jk_{0}x_{1}}$$

(10) $$p_{2}={\hat{p}}_{I}e^{jk_{0}x_{2}}+{\hat{p}}_{R}e^{-jk_{0}x_{2}}$$

where ${\hat{p}}_{I}$ and ${\hat{p}}_{R}$ are the amplitudes of acoustic pressures for the incident (I) and reflected (R) acoustic waves.

Frequency dependencies of the normal-incidence sound reflection coefficient *β* of the tested 3D-printed PLA material samples were measured using a two-microphone acoustic impedance tube (BK 4206) in combination with a power amplifier (BK 2706) and a signal PULSE multi-analyzer (BK 3560-B-030) in the frequency range from 200 to 6400 Hz (Brüel & Kjær, Nærum, Denmark). A circuit diagram of the experimental equipment is depicted in Figure 3a. Normal-incidence sound reflection properties of the tested 3D-printed open-porous specimens of a given thickness *t* (i.e., 10, 20 and 30 mm) and a total volume porosity *P* (from 30 to 56%) were experimentally measured for different air gap sizes *a* (ranging from 0 to 100 mm) behind the studied PLA samples (see Figure 3b). Here, the solid wall SW is characterized by the perfect reflectivity (i.e., *β* = 1) of acoustic waves from its surface. All measurements were performed at an ambient temperature of 22 °C.

## 3. Results and Discussion

This section discusses various factors that influenced the sound reflection properties of the studied 3D-printed open-porous PLA structures.

### 3.1. Influence of Structure Type

**Figure 4:**
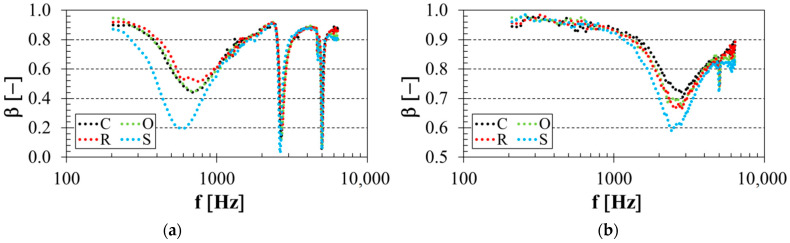
Influence of 3D-printed PLA structure type on the frequency dependencies of the sound reflection coefficient; (**a**) porosity *P* = 30%, specimen thickness *t* = 10 mm, air gap size *a* = 70 mm, (**b**) porosity *P* = 56%, specimen thickness *t* = 10 mm, air gap size *a* = 15 mm.

The material structure is an important factor that has a strong influence on sound reflection performance. The effect of the structure type on sound reflection properties of the tested 3D-printed open-porous PLA materials, which was significant for thin (i.e., *t* = 10 mm) PLA material samples, is demonstrated in Figure 4. The frequency dependencies of the sound reflection coefficient for the PLA specimens of porosity *P* = 30% and thickness *t* = 10 mm are shown in Figure 4a. In this instance, the specimens were placed at 70 mm from the solid wall SW inside the acoustic impedance tube (see Figure 3b). Similarly, the effect of the structure type of the tested open-porous PLA materials (with *P* = 56%, *t* = 10 mm and *a* = 15 mm) is demonstrated in Figure 4b. It is clear from Figure 4 that lower sound reflection properties were generally found for the PLA samples manufactured with the starlit structure, which were characterized by more complex pore shapes compared to the other open-porous PLA structures. For this reason, the PLA samples produced with the starlit structure exhibited higher airflow resistivity, which was accompanied by higher internal friction during the propagation of sound waves through this specimen structure, and by higher conversion of the incident acoustic energy *E_I_* (see Figure 3b) into heat. These findings were observed mainly for low-frequency sound waves.

It can be concluded that the sound reflection performance for the investigated 3D-printed open-porous PLA materials of thickness *t* = 10 mm was significantly influenced by their volume porosity. In the case of low-porous (i.e., *P* = 30%) PLA materials, better sound reflection behavior was observed for the specimens made with the rhomboid structure (see Figure 4a). However, the PLA materials of thickness *t* = 10 mm produced with the cartesian structure exhibited better sound reflection performance for the highest porosity value (i.e., *P* = 56%), as shown in Figure 4b. The influence of the structure type on the sound reflection behavior of the tested 3D-printed open-porous PLA materials was negligible for the thicker (i.e., *t* = 20 and 30 mm) materials.

### 3.2. Influence of Total Volume Porosity

**Figure 5:**
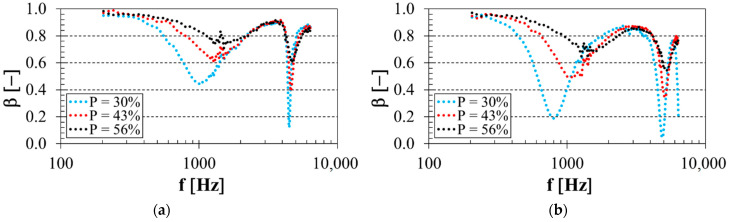
Effect of total volume porosity on the frequency dependencies of the sound reflection coefficient for the investigated PLA specimens: (**a**) rhomboid structure, sample thickness *t* = 10 mm, air gap size *a* = 40 mm; (**b**) cartesian structure, sample thickness *t* = 20 mm, air gap size *a* = 30 mm.

Sound reflection properties of the investigated four different open-porous PLA structures were significantly influenced, not only by the pore shape, but also by its size. It is known that the total volume porosity of open-porous materials is proportional to their relative density that increases with the decreasing pore size [56]. Better sound absorption properties of open-porous material structures are generally obtained at higher material density and airflow resistivity and smaller average pore sizes [57,58]. For these reasons, the increasing porosity of open-porous material structures leads to better sound reflection properties of these materials. This phenomenon was confirmed by the measured frequency dependencies of the sound reflection coefficient, as shown in Figure 5. Figure 5a represents the porosity effect on sound reflection performance of the PLA sample, which was manufactured with the rhomboid structure and thickness *t* = 10 mm and was placed at distance *a* = 40 mm in front of the solid wall SW. Similarly, the sound reflection properties of the PLA sample with the cartesian structure, thickness *t* = 20 mm and air gap size *a* = 30 mm depending on its porosity, are depicted in Figure 5b. It is clear from Figure 5 that the sound reflection coefficient generally increased with increasing sample porosity, especially at low excitation frequencies. This is because the higher porosity resulted in lower airflow resistivity during the sound wave propagation through the PLA open-porous structures, resulting in better sound reflection properties.

### 3.3. Influence of Specimen Thickness

**Figure 6:**
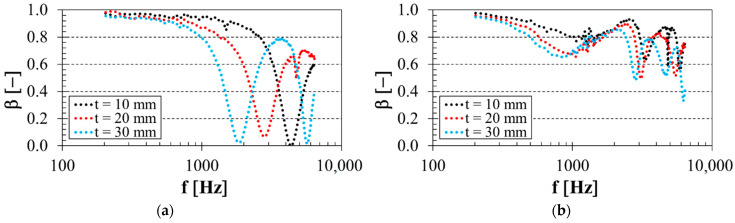
Effect of specimen thickness on the frequency dependencies of the sound reflection coefficient for the investigated PLA specimens: (**a**) Starlit structure, porosity *P* = 30%, air gap size *a* = 0 mm; (**b**) Octagonal structure, porosity *P* = 56%, air gap size *a* = 60 mm.

The thickness of the 3D-printed open-porous PLA materials was also an important factor affecting their sound reflection behavior. The effect of the specimen thickness on the frequency dependencies of the sound reflection coefficient is shown in Figure 6. Figure 6a demonstrates the effect of specimen thickness on the sound reflection performance of the PLA sample with 30% porosity that was made with the starlit structure and was placed directly on the solid wall SW (i.e., *a* = 0 mm). Similarly, the influence of the thickness of the PLA sample with the highest porosity (i.e., *P* = 56%), which was produced with the octagonal structure and was placed 60 mm from the solid wall SW inside the acoustic impedance tube, is demonstrated in Figure 6b. It is clear that the sound reflection properties of the tested PLA samples decreased with increasing material thickness in a substantial part of the measured frequency range, especially in the low-frequency region. This phenomenon was caused by higher internal friction during the sound wave propagation through the thicker open-porous PLA materials, which was reflected in a greater conversion of sound wave energy into heat. Therefore, the increasing thickness of open-porous materials generally reduced their sound reflection properties. Lower sound reflection performance was found for the thicker PLA materials at the excitation frequency *f* ≅ 3 kHz depending on the structure type, the volume porosity *P* and the air gap size *a*. This frequency boundary generally decreased with decreasing sample volume porosity and increasing air gap size.

### 3.4. Influence of Air Gap Size

**Figure 7:**
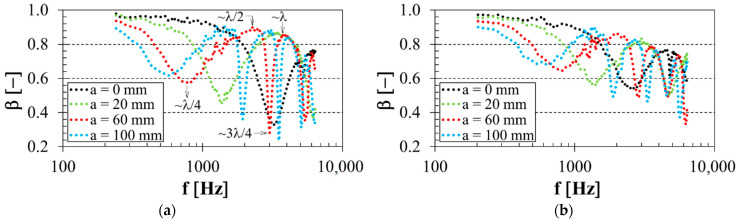
Influence of air gap size on the frequency dependencies of the sound reflection coefficient for the studied PLA specimens: (**a**) Starlit structure, sample thickness *t* = 20 mm, porosity *P* = 43%; (**b**) Cartesian structure, sample thickness *t* = 20 mm, porosity *P* = 56%.

The sample distance in front of the solid wall SW inside the acoustic impedance tube was also a significant factor influencing the sound reflection properties of the investigated 3D-printed open-porous PLA materials. It is clear from Figure 7 that the frequency dependencies of the sound reflection coefficient were periodic with a certain number of minima and maxima of the sound reflection coefficient over the whole measured frequency range. This phenomenon, characteristic of open-porous materials, was demonstrated by the sound wave reflection from the wall SW inside the acoustic impedance tube (see Figure 3b), which relates to the wavelength of sound *λ*. The acoustic pressure will be highest at the wall, but the air particle velocity will be zero because the sound waves cannot supply enough energy to shake the solid wall [59]. If the air gap size between the solid wall and the tested sample gradually increases, the acoustic pressure decreases, but the air particle velocity increases. When the air gap is set to correspond to a quarter wavelength, the acoustic pressure is zero, and the air particle velocity is maximal. It is subsequently accompanied by higher internal friction during the propagation of sound waves through the porous specimen structure, which led to low sound reflection properties in this case. However, placing the open-porous material at a half-quarter wavelength distance from the solid wall will have a maximum reflection effect because the air particle velocity is minimum, and the acoustic pressure is maximum. Therefore, the sound reflection minima are proportional to odd multiples of quarter wavelengths and are obtained at the excitation frequencies:

(11) $$f_{min}=\frac{c\cdot \left(2n+1\right)}{4l}=\frac{c\cdot \left(2n+1\right)}{4\cdot \left(a+\frac{t}{2}\right)}$$

where *c* is the speed of sound, *n* is the integer (*n* = 0, 1, 2…), and *l* is the distance between the studied sample and the solid wall inside the acoustic impedance tube. Similarly, the sound reflection maxima are proportional to even multiples of quarter wavelengths and are obtained at the excitation frequencies:

(12) $$f_{max}=\frac{c\cdot n}{2l}=\frac{c\cdot n}{2\cdot \left(a+\frac{t}{2}\right)}$$

It can be seen from Figure 7 that the excitation frequencies corresponding to sound reflection minima and maxima generally decreased with increasing air gap size, which is consistent with Equations (11) and (12) above. For this reason, the air gap size had a negative effect on sound reflection performance, especially in the low-frequency region. It is also clear that increasing air gap size led to a higher number of frequency minima and maxima over the whole measured frequency range. 

Examples of the experimentally obtained values of the primary sound reflection minima and maxima, and their corresponding excitation frequencies, are shown in Table 2 and Table 3. Table 2 demonstrates the measured values of the primary sound reflection minima *β_min_*_1_ (proportional to a quarter-wavelength, i.e., *λ*/4), the primary sound reflection maxima *β_max_*_1_ (proportional to a half-wavelength, i.e., *λ*/2) and the relevant frequencies *f_min_*_1_ and *f_max_*_1_ depending on the air gap size *a* of the most porous (i.e., *P* = 56%) PLA samples measuring 10 mm in thickness. It is obvious that the frequencies, which correspond to the primary sound reflection minima and maxima, generally shifted to lower values of the excitation frequencies with the increasing air gap size. However, the primary sound reflection minima and maxima generally increased with increasing air gap size. It can also be seen that the lowest values of the primary sound reflection minima and maxima and their corresponding excitation frequencies were found for the PLA samples produced with the starlit structure, which is in accordance with Figure 4. Similar results were found for the low porous (i.e., *P* = 30%) samples measuring 30 mm in thickness, as shown in Table 3. Here, the increasing air gap size led to an increase in the primary sound reflection minima and maxima and to a decrease in their corresponding excitation frequencies. However, it is also clear that the effect of the structure type of the thicker PLA samples on these excitation frequencies was practically negligible compared to the thin samples tested (see Table 2). Again, it was found that lower values of the sound reflection coefficient were observed for the specimens that were manufactured with the starlit structure. This was due to the more complex pore shapes of this 3D-printed structure than the other open-porous PLA structures, which led to multiple reflections during the propagation of sound waves through the starlit structure and consequently to greater conversion of sound energy into heat.

Placing the porous sample at a suitable distance from the solid wall is one way to ensure higher sound reflectivity while reducing weight. Applying open-porous materials with an air gap size to reflect sound is advantageous, especially at higher excitation frequencies, because porous (e.g., fibrous and foam) materials exhibit generally good sound absorption properties at high frequencies [60,61,62,63,64]. Furthermore, 3D-printed open-porous PLA material structures are characterized by a relatively high stiffness/weight ratio, resulting in lower production costs.

### 3.5. Influence of Excitation Frequency

As stated above in Figure 4, Figure 5, Figure 6 and Figure 7, the sound reflection properties of the investigated 3D-printed PLA specimens were also strongly influenced by the excitation frequency. It can be seen that good sound reflection properties were generally observed in a low-frequency range, whose upper frequency limit generally increased with increasing sample porosity and with decreasing air gap size and specimen thickness. In the case of higher excitation frequencies, better sound reflectivity can be achieved by appropriately adjusting the air gap size behind the given 3D-printed PLA sample inside the impedance tube. From Equation (12) mentioned above, the appropriate size of this air gap in the desired frequency range can be determined as follows:

(13) $$a=\frac{c\cdot n}{2f}-\frac{t}{2}$$

### 3.6. Comparison of Sound Reflection Properties

The ability of the investigated 3D-printed open-porous PLA materials to reflect sound was compared using the mean value of the sound reflection coefficient *β_m_* that was determined as an arithmetical average of the sound reflection coefficients over the whole measured frequency range (i.e., from 200 to 6400 Hz). Some examples of the calculated values of the mean sound reflection coefficient are shown in Table 4. Regardless of the structure type of the tested porous PLA materials, it is obvious that the mean value of the sound reflection coefficient generally increased with increasing sample porosity and with decreasing specimen thickness and air gap size behind the tested sample inside the impedance tube. It was confirmed that the PLA specimens manufactured with the starlit structure exhibited the lowest sound reflection properties because of their complex shape structure compared to the other types of studied open-porous PLA structures. It can also be seen that the sound reflection behavior of the PLA samples, which were produced with the other types (i.e., cartesian, octagonal and rhomboid) of open-porous structures, was very similar. The highest value of the mean sound reflection coefficient (i.e., *β_m,max_* = 0.857), and thus the highest ability to reflect sound, was found for the PLA specimen that was produced with the cartesian structure, the highest total volume porosity (i.e., *P* = 56%), the smallest thickness (i.e., *t* = 10 mm) and which was placed on the solid wall SW (i.e., *a* = 0 mm).

## 4. Summary

It can be concluded that the sound reflection properties of the investigated lightweight 3D-printed PLA specimens were significantly influenced by many factors, namely, their volume porosity and thickness, the structure type, the air gap size behind the tested samples and the excitation frequency. In general, good sound reflection properties were obtained using hard, rigid, and non-porous materials (e.g., concrete, stone, and metals) and low excitation frequencies. Conversely, soft, and porous materials were characterized by high soundproofing properties, mainly at high excitation frequencies. The application of thin, lightweight, open-porous 3D-printed PLA materials in the high-frequency region presents a new perspective in terms of sound reflection compared to classic rigid, heavy materials. Based on the experimental data of the investigated open-porous 3D-printed PLA specimens, better sound reflection properties can be obtained by appropriately adjusting the air gap size behind the thin specimens at a given excitation frequency. Therefore, the application of thin, lightweight 3D-printed materials is promising in terms of material weight reduction and energy savings. In future, it will be possible to develop advanced lightweight 3D-printed material structures, which cannot be produced by conventional manufacturing technologies, in order to reflect sound.

## 5. Conclusions

3D printing is a developing technology affecting many areas of life. 3D printing technology allows the production of lightweight materials of different shapes and structures compared to other manufacturing technologies, which leads to time and energy savings and reduces the weight of materials.

The purpose of this study was to investigate the sound reflection performance of 3D-printed open-porous PLA materials made with four different types of porous structures. In addition, the investigated PLA specimens were manufactured with different porosities and thicknesses.

It was found that the sound reflection properties of the studied PLA specimens were influenced, not only by the type of 3D-printed structure, but also by the volume porosity and thickness of the material samples and the air gap size behind the investigated PLA specimens inside the acoustic impedance tube. It can be concluded that the PLA samples manufactured with the starlit structure exhibited lower sound reflection compared to the other investigated PLA structures, especially for specimens with a thickness of 10 mm. This was because the starlit structure is created by more complex pore shapes, resulting in multiple reflections during the propagation of soundwaves through this structure and thus greater conversion of sound energy into heat. It can also be stated that a higher ability to reflect sound from the tested 3D-printed open-porous PLA materials was generally observed at low excitation frequencies and for thin, highly porous samples without the air gap size inside the impedance tube. At higher excitation frequencies, it was possible to place the porous lightweight sample at a suitable distance from the solid wall to improve the sound reflectivity while reducing its weight compared to hard, unperforated solids, which are generally characterized by good sound reflection properties. For this reason, it is possible, for these purposes, to save time, production costs and energy, which is one of the current worldwide trends.

## Figures and Tables

**Table 1:** Basic characteristics of lattice structures designed to investigate the sound reflection properties.

Structure Type	Label	Volume Porosity (%)	Structure View	Strut Diameter (mm)	Cell Size *x/y/z* (mm)
Cartesian		56	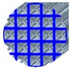	1	5/5/5
	C	43		1.4	5/5/5
		30		1.8	5/5/5
Octagonal		56	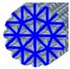	1	6/7/5
	O	43		1.4	6/7/5
		30		1.7	5.5/7/5
Rhomboid		56	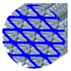	1	5.5/7/7
	R	43		1.35	5/7/7
		30		1.7	5/7/7
Starlit		56	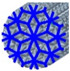	1	8/9/5
	S	43		1.4	7.5/9/5
		30		1.8	8/9/5

**Table 2:** Primary sound reflection minima and maxima and their corresponding excitation frequencies depending on the air gap size for the studied 3D-printed open-porous PLA material structures of thickness *t* = 10 mm and porosity *P* = 56%.

Structure Type	*a* (mm)	*f_min_*_1_ (Hz)	*β_min_*_1_ (-)	*f_max_*_1_ (Hz)	*β_max_*_1_ (-)
Cartesian	25	2272	0.73	5904	0.91
	50	1392	0.75	2984	0.94
	70	1192	0.78	2296	0.95
	100	784	0.81	1424	0.97
Octagonal	25	1936	0.70	5032	0.91
	50	1336	0.74	2920	0.93
	70	1040	0.76	2256	0.93
	100	728	0.81	1360	0.97
Rhomboid	25	2024	0.71	5536	0.90
	50	1384	0.72	2960	0.91
	70	1080	0.77	2272	0.93
	100	760	0.81	1392	0.98
Starlit	25	1928	0.60	5024	0.89
	50	1320	0.64	2872	0.92
	70	960	0.70	2224	0.92
	100	680	0.75	1248	0.92

**Table 3:** Primary sound reflection minima and maxima and their corresponding excitation frequencies depending on the air gap size for the studied 3D-printed open-porous PLA material structures of thickness *t* = 30 mm and porosity *P* = 30%.

Structure Type	*a* (mm)	*f_min_*_1_ (Hz)	*β_min_*_1_ (-)	*f_max_*_1_ (Hz)	*β_max_*_1_ (-)
Cartesian	0	1728	0.00	3608	0.79
	25	704	0.09	2056	0.86
	50	488	0.14	1760	0.87
	100	336	0.18	1288	0.91
Octagonal	0	1776	0.01	3672	0.79
	25	712	0.10	2152	0.85
	50	488	0.16	1808	0.86
	100	336	0.20	1312	0.91
Rhomboid	0	1864	0.02	3696	0.79
	25	728	0.14	2176	0.86
	50	512	0.19	1824	0.86
	100	360	0.24	1344	0.88
Starlit	0	1824	0.02	3640	0.80
	25	696	0.08	2120	0.84
	50	504	0.13	1792	0.84
	100	344	0.20	1296	0.86

**Table 4:** Mean values of the sound reflection coefficient of selected 3D-printed open-porous PLA materials.

Structure Type	*t* (mm)	*P* (%)	*a* (mm)	*β_m_* (-)
Cartesian	10	56	0	0.857
	20	43	50	0.702
	30	30	100	0.598
Octagonal	10	56	0	0.844
	20	43	50	0.699
	30	30	100	0.601
Rhomboid	10	56	0	0.842
	20	43	50	0.692
	30	30	100	0.610
Starlit	10	56	0	0.820
	20	43	50	0.679
	30	30	100	0.589

## Data Availability

Not applicable.
